# The Aquaporin 5 −1364A/C Promoter Polymorphism Is Associated With Cytomegalovirus Infection Risk in Kidney Transplant Recipients

**DOI:** 10.3389/fimmu.2019.02871

**Published:** 2019-12-05

**Authors:** Tim Rahmel, Hartmuth Nowak, Sandra Frisenda, Katharina Rump, Björn Koos, Peter Schenker, Richard Viebahn, Michael Adamzik, Lars Bergmann

**Affiliations:** ^1^Klinik für Anästhesiologie, Intensivmedizin und Schmerztherapie, Universitätsklinikum Knappschaftskrankenhaus Bochum, Bochum, Germany; ^2^Klinik für Chirurgie, Universitätsklinikum Knappschaftskrankenhaus Bochum, Bochum, Germany

**Keywords:** *AQP5*, single nucleotide polymorphism (SNP), cytomegalovirus, immunosuppression, infection risk, kidney transplantation

## Abstract

**Background:** The aquaporin 5 (*AQP5)* −1364A/C promoter single nucleotide polymorphism affects key mechanisms of inflammation and immune cell migration. Thus, it could be involved in the pathogenesis of cytomegalovirus infection. Accordingly, we tested the hypothesis that the *AQP5* promoter −1364A/C polymorphism is associated with the risk of cytomegalovirus infection in kidney transplantation recipients.

**Methods:** We included 259 adult patients who received a kidney transplant from 2007 and 2014 in this observational study. Patients were genotyped for the *AQP5* promoter −1364A/C single nucleotide polymorphism and followed up for 12 months after transplantation. Kaplan–Meier plots and multivariable proportional hazard analyses were used to evaluate the relationship between genotypes and the incidence of cytomegalovirus infection.

**Results:** The incidences of cytomegalovirus infection within 12 months after kidney transplantation were 22.9% for the AA genotypes (43/188) and 42.3% for the AC/CC genotypes (30/71; *p* = 0.002). Furthermore, multivariable COX regression revealed the C-allele of the *AQP5* −1364A/C polymorphism to be a strong and independent risk factor for cytomegalovirus infection. In this analysis, AC/CC subjects demonstrated a more than 2-fold increased risk for cytomegalovirus infection within the first year after kidney transplantation (hazard ratio: 2.28; 95% CI: 1.40–3.73; *p* = 0.001) compared to that in individuals with homozygous AA genotypes.

**Conclusions:** With respect to opportunistic cytomegalovirus infections (attributable to immunosuppression after kidney transplantation), the C-allele of the *AQP5* −1364A/C promoter polymorphism is independently associated with an increased 12-months infection risk. These findings emphasize the importance of genetic variations as additional risk factors of cytomegalovirus infection after solid organ transplantations and might also facilitate the discovery of novel therapeutic targets.

## Introduction

Cytomegalovirus (CMV) is one of the most common opportunistic infections in kidney transplant recipients, which affects transplant rejection and graft function, triggers harmful CMV-associated diseases, and might also influence mortality rates (1, 2). Antiviral chemoprophylaxis seems to be a successful strategy in preventing major complications related to CMV infections, but universal prophylaxis is also detrimental, due to drug toxicity, late CMV disease, and the development of ganciclovir-resistant mutants (3). Therefore, risk-adapted strategies appear to be a cornerstone of modern antiviral chemoprophylaxis and identifying associated risk factors seems to be crucial to improve current post-transplantation care. In this context, the incidence of CMV infections is highly dependent on the serostatus of the recipient (R) and the donor (D) with the highest risk noted in D positive and R negative (D^+^/R^−^) transplantations (4). However, CMV infection risk cannot be solely attributed to this single risk factor, as some of additional variability might be caused by genetic variations (5).

An interesting candidate for investigations regarding such genetic variations is the single nucleotide polymorphism (SNP; rs3759129) in the aquaporin 5 (*AQP5*) promoter region (−1364A/C). Previously, we described that the substitution of cytosine for adenine at position −1364 is associated with lower AQP5 messenger RNA and protein expression (6). In this context, AQP5 expression mediates water transport across biologic membranes, regulating cellular water fluid homeostasis during inflammation, proliferation, and cell migration, processes that involve the transient formation of membrane protrusions (lamellipodia and membrane ruffles) at the leading edge of the cell (7–9). The crucial effect of this *AQP5* SNP in mediating key mechanisms of inflammation and altering related host–pathogen communication was demonstrated in patients with sepsis and acute respiratory distress syndrome (10–12). In this regard, the *AQP5* −1364A/C promoter SNP was found to affect neutrophil migration into the lungs and the AA genotypes were associated with aggravated pulmonary inflammation in acute respiratory distress syndrome evoked by bacteria (10). Strikingly, increased *AQP5* expression and the AA genotype of the *AQP5* SNP were also shown to be associated with improved bacterial eradication, and therefore an enhanced antimicrobial immune response (10, 13).

Taken together, this *AQP5* polymorphism could contribute to the risk of CMV infection in kidney transplant recipients due to an altered resistance to viral infections, but data addressing this topic are completely lacking. Accordingly, we tested the hypothesis that the *AQP5* promoter −1364A/C polymorphism is associated with the risk of CMV infection in kidney transplantation recipients.

## Materials and Methods

### Patients and Treatments

This study was reviewed and approved by the local ethics board of the Faculty of Medicine, Ruhr-University of Bochum (Bochum, Germany; protocol no. 4870-13). Patients were enrolled in this study upon receiving a kidney or combined pancreas–kidney transplant between 2007 and 2014 at the Department of General Surgery of the University Hospital Knappschaftskrankenhaus Bochum (Bochum, Germany). For study inclusion written informed consent was obtained from all 259 participating patients, according to the Declaration of Helsinki, good clinical practice guidelines and applicable to local regulatory requirements.

Patients were recruited to donate a buccal swab for DNA extraction and the evaluation of *AQP5* SNPs after transplantation. Clinical and demographic data were gathered upon study inclusion and patients were observed for 1 year after organ transplantation. All patients received immunosuppressive induction and maintenance therapy according to locally specific standard operating procedures, which included steroids, calcineurin inhibitors, and mycophenolic acid (Table 1), as well as risk-adapted perioperative and post-operative antiviral chemoprophylaxis with ganciclovir or valganciclovir. In this context, 59 high-risk patients (D^+^/R^−^) received chemoprophylaxis for 6 months (except five patients in this group with unknown or shorter duration), 144 medium-risk patients (D^+^/R^+^ and D^−^/R^+^) received prophylaxis for 3 months (except 10 patients in this group with unknown or shorter duration), and 41 low-risk patients (D^−^/R^−^) received perioperative prophylaxis, for whom chemoprophylaxis was expanded to 3 months in 20 cases, for example, due to CMV-positive blood transfusions.

**Table 1:** Characteristics of kidney transplantation patients (*n* = 259) at baseline stratified by *AQP5* −1364 A/C genotype.

**Variable**	**AA** ***n* = 188 (73%)**	**AC/CC** ***n* = 71 (27%)**	***P*-value**
Age (y), mean (range/±SD)	53.3 (23–89/±12.6)	53.0 (28–77/±11.3)	0.890
Male sex, *n* (%)	120 (63.8%)	45 (63.4%)	0.908
Body mass index (*kg/m^2^*), mean (± SD)	25.7 (± 4.4)	26.2 (± 4.6)	0.404
Ethnicity, *n* (%)			1.000
Caucasian	184 (97.9%)	70 (98.6%)	
Other	4 (2.1%)	1 (1.4%)	
Etiology of end-stage renal disease, n (%)			0.675
Glomerular disease	49 (26.1%)	14 (19.7%)	
Diabetes	45 (23.9%)	21 (29.6%)	
Hypertension	21 (11.2%)	6 (8.5%)	
Polycystic kidney disease	25 (13.3%)	12 (16.9%)	
Other/unknown	48 (25.5%)	18 (25.3%)	
Pre-transplantation renal replacement therapy, n (%)	166 (88.3%)	63 (88.7%)	0.922
Transplantation, n (%)			0.908
Kidney	131 (69.7%)	50 (70.4%)	
Combined pancreas + kidney	57 (30.3%)	21 (29.6%)	
Cold ischemia time (min), mean (±SD)	688 (± 315)	674 (± 262)	0.736
First kidney transplantation, *n* (%)	173 (92.0%)	62 (87.3%)	0.245
Previous kidney transplantation, *n* (%)	15 (8.0%)	9 (12.7%)	
HLA-mismatches, median (IQR)	3 (2:5)	4 (2:5)	0.731
0–1, *n* (%)	21 (11.2%)	13 (18.3%)	0.283
2–4, *n* (%)	109 (57.9%)	33 (46.5%)	
≥5, *n* (%)	46 (24.5%)	21 (29.6%)	
Missing, *n* (%)	12 (6.4%)	4 (5.6%)	
Donor			0.558
Age (y), mean (range/± SD)	52.4 (4–85/± 16.3)	49.1 (8–87/± 18.6)	
Male sex, *n* (%)	92 (48.9%)	41 (57.7%)	0.130
Living donor, *n* (%)	21 (11.2%)	10 (14.1%)	0.519
Cadaveric donor, *n* (%)	167 (88.8%)	61 (85.1%)	
Delayed graft function, *n* (%)	52 (27.7%)	23 (32.4%)	0.454
eGFR 1-year after transplantation (ml/min/1.73 m^2^), median (IQR)	46.4 (32.9:59.1)	47.1 (29.8:57.7)	0.613
Biopsy-proven acute rejection, *n* (%)	57 (30.3%)	22 (30.9%)	0.917
Induction with ATG, *n* (%)	155 (82.4%)	56 (78.9%)	0.509
Initial immunosuppressive regimen, *n* (%)			0.684
MPA, prednisone, and tacrolimus	171 (91.0%)	62 (87.3%)	
MPA, prednisone, and cyclosporine	13 (6.9%)	7 (9.9%)	
Other	4 (2.1%)	2 (2.8%	
Usage of mTOR inhibitors, *n* (%)	30 (16.0%)	7 (9.9%)	0.239
CMV infection, *n* (%)	43 (22.9%)	30 (42.3%)	0.002
Time of transplantation to CMV infection (days), median (IQR)	169 (106:265)	115 (70:188)	0.012
CMV disease, *n* (%)	10 (5.3%)	11 (15.5%)	0.007
CMV pneumonia	0	2 (18.2%)	
CMV syndrome	6 (60.0%)	4 (56.3%)	
CMV gastrointestinal disease + hepatitis	4 (40.0%)	2 18.2%)	
Other	0	3 (27.3%)	
Indication of anti-CMV therapy, *n* (%)			0.776
Prophylactic–perioperative	21 (11.2%)	8 (11.3%)	
Prophylactic−3 months	123 (65.4%)	42 (59.1%)	
Prophylactic−6 months	40 (21.3%)	19 (26.8%)	
None/unknown	4 (2.1%)	2 (2.8%)	
Anti-CMV therapy, *n* (%)			0.867
Ganciclovir	18 (9.6%)	8 (11.3%)	
Valganciclovir	166 (88.3%)	61 (85.9%)	
None/unknown	4 (2.1%)	2 (2.8%)	
CMV serology at transplantation, n (%)			0.973
D^+^/R^−^	45 (23.9%)	19 (26.8%)	
D^+^/R^+^	68 (36.2%)	25 (35.2%)	
D^−^/R^+^	45 (23.9%)	16 (22.5%)	
D^−^/R^−^	30 (16.0%)	11 (15.5%)	

*IQR, Interquartile Range with 25th and 75th percentile; HLA, human leukocyte antigen; eGFR, Glomerular filtration rate was estimated by using Modification of Diet in Renal Disease (MDRD) study equation; ATG, antithymocyte globulin; MPA, mycophenolic acid; mTOR, mechanistic target of rapamycin; CMV, cytomegalovirus; D^+^, CMV sero-positive donor; D^−^, CMV sero-negative donor; R^+^, CMV sero-positive recipient; R^−^, CMV sero-positive recipient. Missing data were excluded from the analysis: six cases were missing for body mass index and one case was missing for cold ischemia time*.

Routine surveillance for viral reactivation or infection comprised weekly determinations of CMV viremia based on whole blood samples via PCR, until hospital discharge from index-admission and continuing monthly thereafter and when clinically indicated. Additionally, all patients were screened for CMV infection at the 1-year follow up examination after transplantation. Delayed graft function was defined as the necessity for dialysis in the first week after surgery.

### DNA Genotyping

DNA was extracted from buccal swabs using the QIAamp DNA Mini Kit (QIAGEN, Hilden, Germany). To genotype the −1364A/C *AQP5* promoter SNP, a nested polymerase chain reaction was performed with the forward AQP5-SE 5′-CCCAGACCAGGGGTAGAAGA-3′, and the reverse AQP5-AS 5′-TCTTCCTGCTAGAAGCCCCT-3′ primers followed by tetra-primer ARMS-PCR with Forward inner primer (A allele): 5′-GAGAGAGACAGAGAGACTAAGACAGCGAA-3′, Reverse inner primer (C allele): 5′-CATTTTCTGTTTTTCCTTCCTGCTTG-3′, Forward outer primer 5′-GACCACATGTAAGAGAGAGAGACATGGA-3′ and Reverse outer primer 5′-CTGTCAGTCAGTCTTTGCAAAACCCTAT-3′ resulting in a 223 base pair fragment for A allele and a 189 base pair fragment for C allele.

### Study Groups and End Points

Study patients were assigned to two groups (AA genotype vs. AC/CC genotype) depending on the −1364A/C SNP in the *AQP5* promoter. The AC and CC genotypes were combined because of the low frequency (3.1%; 8/289) of the CC genotype.

The primary end point was CMV-free survival in the first year after kidney transplantation. The key secondary end point was the effect of chemoprophylaxis duration on the time of CMV infection onset.

### Clinical Definitions and Diagnostics

CMV infection was defined as the detection of viral nucleic acid in accordance to the definition of Ljungman and colleagues (14). CMV DNA was evaluated using a commercially available PCR assay (Roche Ampliprep Assay; Roche Molecular Diagnostics, Pleasanton, CA, USA) as per the manufacturer's instructions and calibrated to the World Health Organization International Standard for Human CMV.

CMV disease and related entities (e.g., CMV pneumonia and CMV syndrome) were defined as the presence of CMV in the blood based on a local assay plus the presence of compatible symptoms as described by Ljungman and colleagues (14).

### Statistical Analysis

The characteristics of patients at baseline (timepoint of transplantation) were reported as percentages for categorical variables and as means with standard deviations (±SD) or medians with interquartile ranges (25th; 75th percentile) for continuous variables, as appropriate. Categorical variables were compared with chi-square or Fisher's exact tests, and continuous variables were compared with a parametric Student's *t*-test or non-parametric Wilcoxon-Mann-Whitney-Test. The *AQP5* −1364A/C SNP distributions were tested for deviations from the Hardy–Weinberg equilibrium (exact two-sided *P*-value; significance value, 0.05). Explorative comparisons based on *AQP5* −1364A/C genotypes (AC/CC vs. AA) were performed for several clinical patient characteristics (Table 1).

CMV infection probabilities were graphically assessed by the Kaplan–Meier method. The log-rank test was used to evaluate the univariate relationship between the *AQP5* −1364A/C genotype and incidence of CMV infection. Next, we performed Cox regression analyses assessing the joint effect of the *AQP5* −1364A/C genotype and potential predictors on CMV-free survival. At first, Cox regression was performed with several models based on a single predictor (Table 3, left column). Thereafter, multiple variable Cox regression was performed with an initial model investigating multiple predictors simultaneously (Table 3, right column). To avoid overfitting, a restricted model with only four predictors was assessed subsequently using only those predictors with a *P*-value 0.05 or lower based on either the single or multiple predictor comparisons (Table 4). Confidence intervals (CI) were calculated with a coverage of 95%. All reported *P*-values were nominal and two-sided with an a priori α error of <0.05. All analyses were performed using SPSS (version 24, IBM, USA); for graphical presentations, GraphPad Prism 7 (Graph-Pad, USA) was used.

## Results

The baseline characteristics of the 259 kidney transplant recipients stratified for the *AQP5* −1364A/C promoter SNP are presented in Table 1. The mean age of the recipients at the time of transplantation was 53.2 ± 12.2 years and most were male (63.7%; 165/259). The observed 1-year CMV infection rate of the entire cohort was 28.1% (73/259) and the median duration of CMV infection onset after transplantation was 150 days [90; 217]. Regarding the distribution of genetic variations according to the Hardy–Weinberg equilibrium of the *AQP5* SNPs, we observed a frequency of 188 for the AA-genotype (expected: *n* = 186), 63 for the AC-genotype (expected: *n* = 67), and eight for the CC-genotype (expected: *n* = 6) in our cohort. Accordingly, no deviation from the Hardy–Weinberg equilibrium was observed (*p* = 0.8475).

In addition, 69.9% (181/259) received a kidney and 30.1% (78/259) received a combined pancreas and kidney transplantation, without statistically significant distribution among AA and AC/CC genotypes (*p* = 0.908; Table 1). Furthermore, we found no evidence of statistically significant associations between the *AQP5* −1364A/C genotypes and age (*p* = 0.890), sex (*p* = 0.908), etiology of end-stage renal disease (*p* = 0.675), rate of pre-transplantation renal replacement therapy (*p* = 0.922), delayed graft function (*p* = 0.454), and CMV serology at transplantation (*p* = 0.973). Cases of ganciclovir-resistant CMV strains were not detected among the study patients.

**Figure 1:**
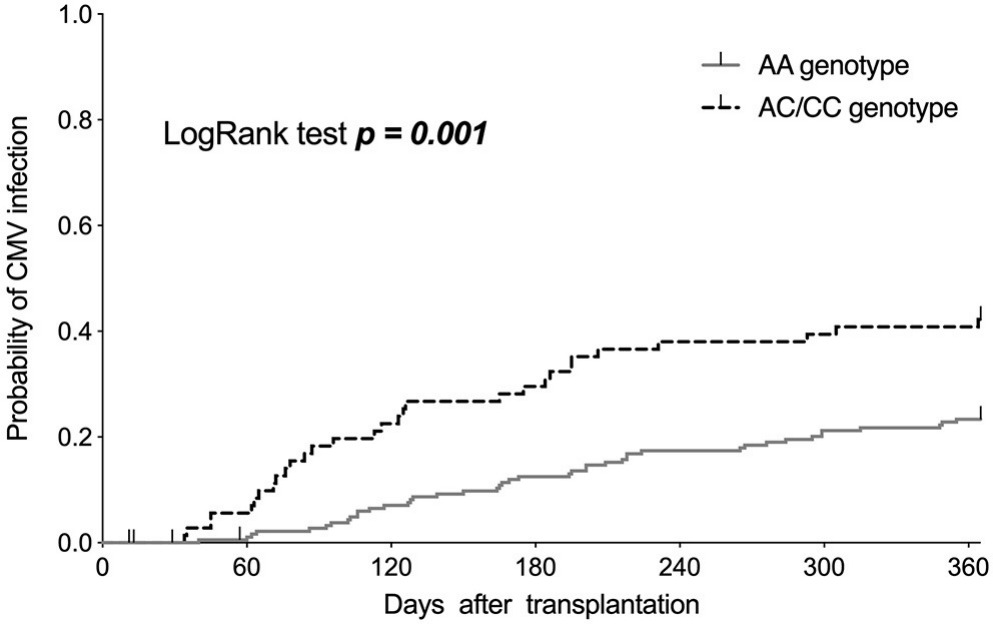
Kaplan–Meier curves showing the incidence of cytomegalovirus (CMV) infections in the first year after kidney transplantation, stratified based on the AA and AC/CC genotypes of the *AQP5* −1364A/C single nucleotide polymorphism.

One-year CMV infection risk was significantly associated with the *AQP5* −1364A/C genotypes (*p* = 0.001; Figure 1). CMV infection rates were 23% (43/188) for the AA genotype and 42% (30/71; *p* = 0.001) for the AC/CC genotypes. In addition, CMV disease was more common in individuals with the AC/CC genotypes (15.5%; 11/71), when compared to the association with the AA genotype (5.3%; 10/188; *p* = 0.007). Further, stratifying patients according to the presence of CMV infections and CMV-associated diseases, there were no statistically significant differences between the AC (39.7%; 25/63 and 14.2%; 9/63, respectively) and CC (62.5%; 5/8; *p* = 0.269 and 25.0%; 2/8; *p* = 0.601, respectively) genotypes (Table 2).

**Table 2:** Characteristics of kidney transplantation recipients (*n* = 259) stratified by frequencies and time of CMV infection onset.

**Variable**	**Total frequencies: *n*_**total**_ (%)**	**Frequency of CMV infection: *n*_**CMV**_ (%)**	**Time to CMV infection, median (IQR)**
*AQP5* −1364A/C Genotype			
AA	188 (72.6%)	43 (22.8%)	169 (106:265)
AC	63 (24.3%)	25 (39.7%)	116 (64:191)
CC	8 (3.1%)	5 (62.5%)	84 (77:238)
*p*-value		*p* = 0.003	*p* = 0.041
Duration of prophylactic Anti-CMV Therapy			
Perioperative, *n* (%)	29 (11.2%)	5 (17.2%)	64 (35:113)
3 months, *n* (%)	165 (63.7%)	41 (24.8%)	126 (94:174)
6 months, *n* (%)	59 (22.8%)	25 (42.4%)	209 (156:289)
None/unknown, *n* (%)	6 (2.3%)	2 (33.3%)	172 (45:299)
*p*-value		*p* = 0.032	*p* = 0.001
CMV Serology at Transplantation, *n* (%)			
D^+^/R^−^	64 (24.7%)	29 (45.3%)	201 (96:280)
D^+/−^/R^+^	154 (59.5%)	39 (25.3%)	126 (93:173)
D^−^/R^−^	41 (15.8%)	5 (12.2%)	103 (61:266)
*p*-value		*p* = 0.001	*p* = 0.073

*CMV, cytomegalovirus; D^+^, CMV sero-positive donor; D^−^, CMV sero-negative donor; R^+^, CMV sero-positive recipient; R^−^, CMV sero-positive recipient*.

Multivariate Cox regression analysis revealed the *AQP5* −1364A/C genotype was both an independent and strong (due to the estimated effect size) risk factor for CMV infection (Tables 3, 4). In this context, C-allele carriers had a more than 2-fold greater risk of CMV infection in the first year after kidney transplantation (hazard ratio 2.28; 95% CI: 1.40–3.73; *p* = 0.001) compared to that with the AA genotype. Furthermore, the D^+^/R^−^ CMV serostatus (hazard ratio 8.61; 95% CI: 2.0–5.7; *p* = 0.003) was confirmed as an important risk factor for CMV infection based on our cox-regression model.

**Table 3:** Univariable and multivariable Cox regression analysis of kidney transplantation recipients regarding the effect on cytomegalovirus infection risk.

**(Co) variable**	**Univariable**			**Multivariable**		
	***p*-value**	**HR**	**95% CI**	***p*-value**	**HR**	**95% CI**
Aquaporin 5 −1364A/C genotype						
AA	–	1		–	1	
AC/CC	0.001	2.196	1.377–3.502	0.001	2.331	1.394–3.899
Recipient age [per year]	0.792	0.997	0.979–1.016	0.400	0.989	0.964–1.015
Recipient sex						
Female	–	1		–	1	
Male	0.989	1.003	0.624–1.614	0.796	1.071	0.637–1.801
Donor age [per year]	0.637	0.997	0.983–1.010	0.533	0.994	0.976–1.013
Donor sex						
Female	–	1		–	1	
Male	0.526	1.160	0.733–1.836	0.768	0.926	0.577–1.540
Cold ischemia time [per h]	0.379	1.020	0.976–1.066	0.353	1.027	0.970–1.088
Transplanted organ						
Kidney	–	1		–	1	
Kidney + pancreas	0.726	1.092	0.667–1.790	0.318	0.703	0.353–1.403
Living donor	–	1		–	1	
Cadaveric donor	0.413	1.385	0.635–3.019	0.339	1.502	0.652–3.458
Delayed graft function [no]	–	1		–	1	
Delayed graft function [yes]	0.513	1.179	0.720–1.932	0.769	1.087	0.622–1.901
BPAR [no]	–	1		–	1	
BPAR [yes]	0.633	1.125	0.694–1.824	0.519	1.188	0.703–2.007
HLA mismatch [per 1]	0.020	1.195	1.028–1.390	0.019	1.234	1.036–1.471
Immunosuppressive regimen						
MPA, prednisone and cyclosporine	–	1		–	1	
MPA, prednisone, and tacrolimus	0.410	0.720	0.330–1.572	0.208	0.590	0.259–1.343
Other	0.909	1.096	0.228–5.278	0.446	2.375	0.257–21.990
CMV risk status						
D^−^/R^−^	–	1		–	1	
D^+/−^/R^+^	0.089	2.241	0.883–5.686	0.109	2.672	0.803–8.892
D^+^/R^−^	0.003	4.248	1.644–10.981	0.004	10.744	2.153–53.628
Agent for anti-CMV prophylaxis						
Ganciclovir	–	1		–	1	
Valganciclovir	0.226	1.866	0.680–5.118	0.605	0.520	0.043–6.221
Prophylactic anti-CMV therapy						
Perioperative	–	1		–	1	
3 months	0.483	1.394	0.551–3.528	0.871	1.193	0.141–10.085
6 months	0.041	2.482	1.017–6.329	0.514	0.493	0.059–4.124

*HR, odds ratio point estimates, 95% CI, and p-values (two-sided) are reported; BPAR, biopsy-proven acute rejection; HLA, human leukocyte antigen; MPA, mycophenolic acid; CMV, cytomegalovirus; D^+^, CMV sero-positive donor; D^−^, CMV sero-negative donor; R^+^, CMV sero-positive recipient; R^−^, CMV sero-positive recipient; six cases with unknown or no prophylactic anti-CMV therapy were excluded from analysis; 16 cases with missing HLA mismatch scores were excluded from analysis; omnibus test of model coefficients: Chi-square 35.9, p = 0.007; Homer-Lemeshow statistics for multivariable approach were as follows: κ^2^ = 7.9; p = 0.445*.

**Table 4:** Restricted multivariable Cox regression analysis of kidney transplantation recipients with respect to the effect on cytomegalovirus infection risk.

**(Co) variable**	**Multivariable-restricted**		
	***p-value***	**HR**	**95% CI**
Aquaporin 5 −1364A/C genotype			
AA	–	1	
AC/CC	0.001	2.282	1.396–3.732
HLA mismatch [per 1]	0.047	1.161	1.002–1.347
CMV risk status			
D^−^/R^−^	–	1	
D^+/−^/R^+^	0.127	2.335	0.787–6.932
D^+^/R^−^	0.003	8.613	2.079–35.685
Prophylactic anti-CMV therapy			
Perioperative	–	1	
3 months	0.676	0.793	0.268–2.347
6 months	0.159	0.359	0.086–1.491

*HR, odds ratio point estimates, 95% CI, and p-values (two-sided) are reported; CMV, cytomegalovirus, D^+^, CMV sero-positive donor; D^−^, CMV sero-negative donor; R^+^, CMV sero-positive recipient; R^−^, CMV sero-positive recipient; six cases with unknown or no prophylactic anti-CMV therapy were excluded from analysis, 16 cases with missing HLA mismatch score were excluded from analysis; omnibus test of model coefficients: Chi-square 29.4, p < 0.001; Homer-Lemeshow statistics for multivariable approach were as follows: κ^2^ = 7.4; p = 0.494*.

**Figure 2:**
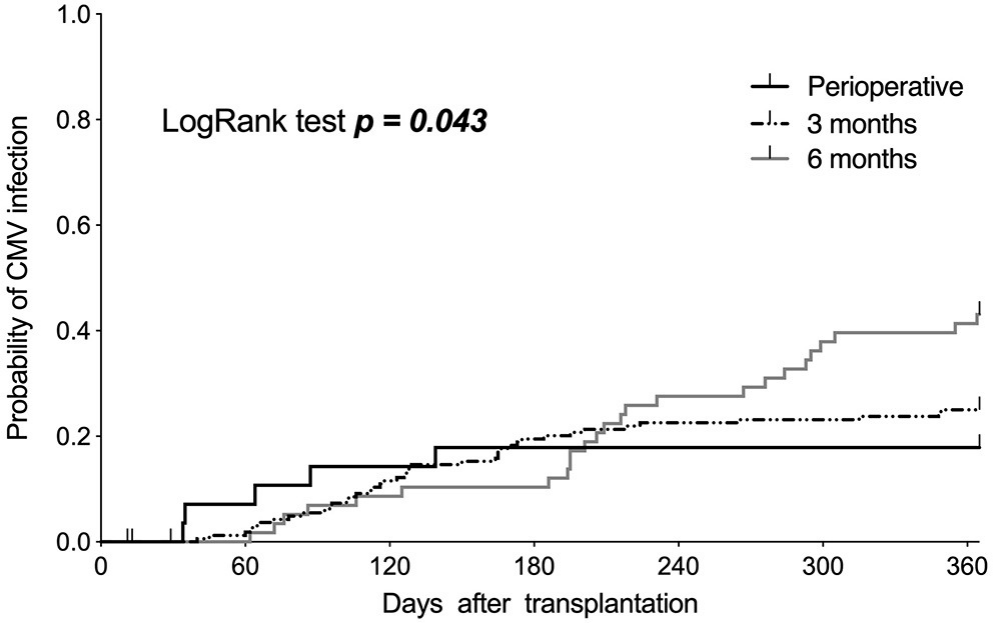
Kaplan–Meier curves showing the incidence of cytomegalovirus (CMV) infections in the first year after kidney transplantation, stratified based on the duration of applied anti-CMV prophylaxis.

Strikingly, the duration of prophylactic anti-CMV therapy did not seem to affect the sustainably of the 1-year CMV infection risk (Table 4, Figure 2). In this context our Kaplan–Meier estimations showed the highest CMV infection rate after 1 year in patients treated with anti-CMV chemoprophylaxis for 6 months (42%, 25/59) compared to that with treatment for 3 months (25%; 41/165) and only perioperative treatment (17.2%; 5/29, *p* = 0.043).

## Discussion

This study shows that the C-allele of the *AQP5* −1364/A/C single nucleotide promoter polymorphism is associated with a marked increase in CMV infection and CMV disease risk in the first year after kidney transplantation. Furthermore, this SNP represents an independent and clinically meaningful risk factor of post-transplant CMV infection, with an estimated hazard ratio of nearly 2.3 for the AC/CC-genotypes. Hence, the *AQP5* −1364A/C promoter SNP might play a pivotal role in the management of post-transplantation CMV prophylaxis.

Since CMV infections continue to have a tremendous effect on outcome in kidney transplant recipients, anti-CMV chemoprophylaxis is a cornerstone of modern post-transplantation management (2). Antiviral prophylaxis involves the administration of antiviral drugs, preferably valganciclovir (15), to all patients at-risk of CMV infection, and is given for up to 6 months, in accordance with the IMPACT trial (16). In this context, the decision on antiviral prophylaxis duration is usually based only on the serostatus of the donor and recipient. However, one major drawback of current antiviral chemoprophylaxis is late-onset CMV infection and disease that is most commonly observed among high-risk CMV D^+^/R^−^ patients after the completion of antiviral prophylaxis (17). This is in line with our results demonstrating a median CMV infection onset time of 129 and 209 days after receiving prophylactic anti-CMV therapy for 3 and 6 months, respectively. Therefore, most cases of CMV infections in patients who received antiviral prophylaxis occur after the cessation of antiviral drug administration, and still predominantly occur in the high-risk D^+^/R^−^ group (17, 18). In this regard, we also found the highest infection rate of 42% 1 year after transplantation, despite the fact that anti-CMV chemoprophylaxis was applied for 6 months in 92% of the D^+^/R^−^ cases. Thus, there seems to be room to further improve the current anti-CMV approaches for post-transplantation management (19, 20). In this context, a recent study elucidates that assessing the cell-mediated anti-CMV immunity could help to identify patients at-risk of developing late-onset CMV infections supporting a guided decision-making to safely stop or better continue antiviral treatment (21). Hence, advances in the field of post-transplantation anti-CMV management will partly be facilitated by the development of first, a better diagnostic assay including genetic variations to the stratify risk of CMV infection, and second, new antiviral agents with unique mechanisms of action and ideally less toxicity.

A promising candidate for further investigation is the common *AQP5* −1364A/C promoter SNP, potentially addressing the aforementioned issues. Obviously, the exact mechanisms associated with genotype-related increased mortality, associated with the AA and AC/CC genotypes, cannot be pinpointed by our study due to absence of profound mechanistical and immunological examinations. However, based on our clinical data and considering previous evidence (8, 22, 23), we speculate that the *AQP5* −1364A/C SNP or rather altered *AQP5* expression might shape the efficiency of immune responses, thereby influencing the efficacy of microbial clearance, and with respect to our study, CMV elimination.

The immune response to CMV infection is highly complex and includes innate and adaptive immune responses (24). Accordingly, CMV infection is first detected by the innate immune system, which seems to be crucial during the early phase of an CMV infection (25). Surprisingly, an important role has been suggested for neutrophils as potent antiviral effector cells that restrict viral replication and associated pathogenesis (26). In this context, it is of note that *AQP5* expression significantly affects the migration and associated activity of neutrophil granulocytes (8, 10). *AQP5*-knockout mice exhibit the attenuated migration of neutrophil granulocytes, which was also associated with higher survival compared to those in wild-type animals after intraperitoneal LPS injection (8). Furthermore, the target-oriented migration of human neutrophils *in vitro* was found to be slower and occurred to a lesser extent with reduced AQP-5 expression. In patients suffering from acute respiratory distress syndrome, attributed to bacterial pneumonia, the AA genotype of the *AQP5* promoter SNP was associated with aggravated pulmonary inflammation accompanied by a significant increase in neutrophil counts in the bronchoalveolar lavage fluid (10). Thus, the AA-genotype of the *AQP5* genotype seems likely to be associated with better neutrophil granulocyte reactivity, which could at least in part explain the lower risk of CMV infection described by this study.

In addition, the sustained control of CMV infection is largely driven by adaptive immunity, involving broadly targeted CMV-specific T-cells to achieve viral control (27). Furthermore, patients with the delayed emergence of CMV-specific CD4^+^ T-helper cells are more likely to develop a CMV infection (28). In addition, evidence from kidney transplantation has confirmed that the frequency of CMV-specific T-helper cells is inversely correlated with the incidences of CMV replication, high CMV load, and onset of CMV-related disease (29–31).

Strikingly, *AQP5* expression also seems to profoundly affect the T-cell response. A recent study demonstrated that T-cell specific cytokines are significantly down-regulated in *AQP5*-knockout mice (32), thus suggesting the crucial contribution of *AQP5* to the effectiveness of T-cell driven immune responses. More recently, the relationship between the *AQP5* deletion and elevated IFN-α and IL-2 production was shown, indicating an effect on the shift from type 2 T-helper cells toward a type 1 phenotype (33). Considering these results, it can be suggested that the *AQP5* −1364A/C promoter SNP critically shapes the innate and adaptive immune response in response to CMV infections. These hypotheses are in line with our results demonstrating that AC/CC genotypes of the *AQP5* −1364A/C SNP are strong and independent risk factors of CMV infection, as compared to the risk with AA genotypes.

Our results could be considered contradictory as the AA genotype of the *AQP5* −1364A/C SNP was found to be associated with worse outcome in our previous studies on sepsis (11) and ARDS (10). In contrast, the present study reports that the AA-genotype can diminish the risk of CMV infection and thus can potentially confer protective effects for kidney transplant recipients. However, sepsis and ARDS are phenomena in which an exaggerated immune response prevails, and therefore, the collateral damage observed with AA genotypes would be in the foreground and caused by a more potent immune system. In kidney transplantation recipients, exactly the opposite must be presumed, because of the profound immunosuppression. In this context, the enhanced immunoreactivity observed with the AA genotypes might mediate immunological benefits in immunosuppressed patients.

Nevertheless, these relationships, and especially the mechanistic associations, must be elucidated in the future, since this approach might also offer a new therapeutic target. In this context, it has been demonstrated that dexamethasone and ambroxol can upregulate AQP-5 expression *in-vitro* (34). Modulating *AQP5* expression depending on the genotype could be an interesting focal point for additional or rather optimized CMV prophylactic strategies. However, whether this approach offers therapeutic or prophylactic benefits, needs to be elucidated in future investigations.

### Limitations

The limitations of this study must also be mentioned. First, unrecognized selection bias, inherent to many genetic association studies, cannot be entirely excluded. Second, our study was almost exclusively conducted on patients of European-Caucasian descent, and therefore, findings cannot be generalized to subjects of other ancestries. Third, although all patients were treated with a rather standardized multimodal regimen, undetected confounding factors might have distorted the results because of the multidimensionality of solid organ transplantation, immunosuppression, and immune responses against CMV infection. However, the single center nature of this study might be an advantage as it limits the varied protocols that can be used when treating kidney transplant recipients. Finally, the observational design, the absence of a reasonable control group, and lack of histologic and mechanistic examinations precludes verification of the causality and underlying mechanisms. Additional studies, especially to uncover mechanistic insights, are needed to further asses the effect of *AQP5* expression on inflammation and immune cell migration, as it relates to CMV infection risk.

### Conclusions

During opportunistic CMV infections attributed to immunosuppression after kidney transplantation, the C-allele of the *AQP5* −1364A/C promoter polymorphism is independently associated with an increased 12-months infection risk. These findings emphasize the importance of genetic variations as additional risk factors of CMV infection after solid organ transplantation, which might also facilitate the discovery of novel therapeutic targets. Consequently, increasing *AQP5* expression in AC and CC genotypes could be an interesting therapeutic approach for organ transplant recipients.

## Data Availability Statement

The raw data supporting the conclusions of this article will be made available by the authors, without undue reservation, to any qualified researcher.

## Author Contributions

TR, HN, PS, RV, MA, and LB: conceived and designed the research. SF, TR, HN, KR, BK, and LB: performed the experiments. KR and BK: contributed the reagents. TR, SF, PS, RV, MA, and LB: collected and provided the clinical data. TR, HN, BK, PS, MA, and LB: interpreted the data. TR, HN, MA, and LB: performed the statistical analysis. TR, HN, PS, and LB: wrote the initial draft. All authors critically revised and approved the manuscript and are accountable for the accuracy and integrity of the work.

### Conflict of Interest

The authors declare that the research was conducted in the absence of any commercial or financial relationships that could be construed as a potential conflict of interest.
