# Corrigendum: The Aquaporin 5 −1364A/C Promoter Polymorphism Is Associated With Cytomegalovirus Infection Risk in Kidney Transplant Recipients

**DOI:** 10.3389/fimmu.2021.739229

**Published:** 2021-08-12

**Authors:** Tim Rahmel, Hartmuth Nowak, Sandra Frisenda, Katharina Rump, Björn Koos, Peter Schenker, Richard Viebahn, Michael Adamzik, Lars Bergmann

**Affiliations:** ^1^Klinik für Anästhesiologie, Intensivmedizin und Schmerztherapie, Universitätsklinikum Knappschaftskrankenhaus Bochum, Bochum, Germany; ^2^Klinik für Chirurgie, Universitätsklinikum Knappschaftskrankenhaus Bochum, Bochum, Germany

**Keywords:** *AQP5*, single nucleotide polymorphism (SNP), cytomegalovirus, immunosuppression, infection risk, kidney transplantation

Sandra Frisenda was not included as an author in the published article. The corrected Author Contributions Statement appears below.

The authors apologize for this error and state that this does not change the scientific conclusions of the article in any way. The original article has been updated.

## Publisher’s Note

All claims expressed in this article are solely those of the authors and do not necessarily represent those of their affiliated organizations, or those of the publisher, the editors and the reviewers. Any product that may be evaluated in this article, or claim that may be made by its manufacturer, is not guaranteed or endorsed by the publisher.

## Author Contributions

TR, HN, PS, RV, MA, and LB: conceived and designed the research. SF, TR, HN, KR, BK, and LB: performed the experiments. KR and BK: contributed the reagents. TR, SF, PS, RV, MA, and LB: collected and provided the clinical data. TR, HN, BK, PS, MA, and LB: interpreted the data. TR, HN, MA, and LB: performed the statistical analysis. TR, HN, PS, and LB: wrote the initial draft. All authors critically revised and approved the manuscript and are accountable for the accuracy and integrity of the work.

